# Innovative tools and OpenHDS for health and demographic surveillance on Rusinga Island, Kenya

**DOI:** 10.1186/s13104-015-1373-8

**Published:** 2015-09-01

**Authors:** Tobias Homan, Aurelio Di Pasquale, Ibrahim Kiche, Kelvin Onoka, Alexandra Hiscox, Collins Mweresa, Wolfgang R. Mukabana, Willem Takken, Nicolas Maire

**Affiliations:** Laboratory of Entomology, Wageningen University and Research Centre, Wageningen, The Netherlands; Department of Epidemiology and Public Health, Swiss Tropical and Public Health Institute, Basel, Switzerland; University of Basel, Basel, Switzerland; Department of Medical Entomology, International Centre of Insect Physiology and Ecology, Nairobi, Kenya; School of Biological Sciences, University of Nairobi, Nairobi, Kenya

**Keywords:** Health and demographic surveillance system, Mobile data collection, Data management platform, Malaria, Kenya

## Abstract

**Background:**

Health in low and middle income countries is on one hand characterized by a high burden associated with preventable communicable diseases and on the other hand considered to be under-documented due to improper basic health and demographic record-keeping. health and demographic surveillance systems (HDSSs) have provided researchers, policy makers and governments with data about local population dynamics and health related information. In order for an HDSS to deliver high quality data, effective organization of data collection and management are vital. HDSSs impose a challenging logistical process typically characterized by door to door visits, poor navigational guidance, conducting interviews recorded on paper, error prone data entry, an extensive staff and marginal data quality management possibilities.

**Methods:**

A large trial investigating the effect of odour-baited mosquito traps on malaria vector populations and malaria transmission on Rusinga Island, western Kenya, has deployed an HDSS. By means of computer tablets in combination with Open Data Kit and OpenHDS data collection and management software experiences with time efficiency, cost effectiveness and high data quality are illustrate. Step by step, a complete organization of the data management infrastructure is described, ranging from routine work in the field to the organization of the centralized data server.

**Results and discussion:**

Adopting innovative technological advancements has enabled the collection of demographic and malaria data quickly and effectively, with minimal margin for errors. Real-time data quality controls integrated within the system can lead to financial savings and a time efficient work flow.

**Conclusion:**

This novel method of HDSS implementation demonstrates the feasibility of integrating electronic tools in large-scale health interventions.

## Background

Health and demographic surveillance systems (HDSS) are used to provide a framework for prospective collection of demographic and public health data within a community. Such systems, originally called population laboratories, have been in operation since the late 20th century, and constitute the basis of population-based research in areas where national or local authorities lack a proper registration system to monitor the most important demographic events [1]. In order for population and health researchers to acquire longitudinal data on communities, systematically constructed systems have undergone several developments [2]; where originally the focus remained on surveying demographic data (demographic surveillance systems, DSS), principally due to efforts of the INDEPTH network (International Network of field sites with continuous Demographic Evaluation of Populations and Their Health in developing countries), health indicators became a routine part of science-driven surveillance systems, retitling the concept as HDSS (health and demographic surveillance system) [3]. Despite these developments, public health systems in developing countries often lack adequate infrastructure to monitor demographic and health information; rural areas in particular experience challenges with the collection of reliable health-related data. The World Health Organization (WHO) states that vast rural areas in Sub-Saharan Africa are a reservoir for a variety of predominantly preventable communicable diseases such as HIV/AIDS, tuberculosis and malaria (WHO; World Health Statistics 2014). The absence of well-operating national or local demographic and health surveillance systems hampers evidence-based research into these diseases. Over the past decades there are numerous examples of scientific institutions deploying community-based HDSSs in order to provide policy makers and governments with recommendations on health planning and intervention methods. A classic example is the Garki project in Nigeria where, during the 1970s, field experiments were conducted to understand the effects of indoor residual spraying (IRS) and mass drug administration (MDA) on malaria and entomological outcomes [4]. Another, more recent, malaria control study which used HDSS to capture prospective data was the Asembo Bay Cohort Project, which ultimately showed a large protective effect of long lasting insecticidial nets (LLIN) against malaria infection.

Nowadays, community-based HDSSs are established at an increasing number of sites to investigate a range of different health indicators and diseases. The main goal of the INDEPTH network is to harmonize the data of HDSSs from different sites in developing countries to achieve a valid comparison of information and accordingly get more insight into health related trends [5].

There are currently 43 INDEPTH associated centres that run one or more HDSSs for scientific purposes [6].

At all these HDSS sites, the field and data management operations pose logistical challenges. Interviews in most sites are essentially paper based which makes conducting questionnaires time consuming and error prone. Visiting households and individuals can be time consuming, as keeping track of where fieldworkers navigate and which community members have been visited can only be done manually. Likewise, transferring data from paper into a digital form is a lengthy process with a lot of room for error. Not only the content of data can be entered incorrectly, but assigning new data to the right entity or ID is an error-prone process with small typos leading to unrecognizable and ultimately squandered data [7–10]. Finally, accumulating and managing data relies heavily on obsolete database software with limited data quality assurance structures.

The past decade has borne witness to major developments in mobile computer technology as well as software applications. Advanced computer tablets and improved data collection and management software have become accessible and affordable to the wider public. In high and middle income countries there are numerous examples of ways to utilize the available technologies to improve health [11, 12]. Although there have been several pilot studies which experimented with a telephone-based technology to collect health and demographic data, in the lower income countries these technologies remain mainly underused because of logistical and organizational constraints [13, 14].

In some low- income countries, mobile computer technology and advanced data collection and management software has been tested. In Akpabuyo Nigeria, the use of computer tablets with practical collection software and a comprehensive data management system has been tested [15]. The study showed that it is possible to save a great deal of time compared to the paper-based and analogue data collection and management. Not only time could be saved, costs could also be decreased considerably and data quality increased. Another study in Malawi investigated how the use of computer technology and software could best be organized to create a feasible system of health data collection and management [16]. A governmental initiative in Kenya in 2006 marked a first step towards a digitalized health management [17].

In 2012 an HDSS was initiated on Rusinga Island, western Kenya, to facilitate a large malaria control trial, the SolarMal project [18]. This paper describes the computer-based HDSS developed for this project. It is shown that community-based health research served by HDSSs can be of higher quality, more cost-effective and more time efficient than currently deployed surveillance systems.

## Methods

### Study location and population

**Fig. 1:**
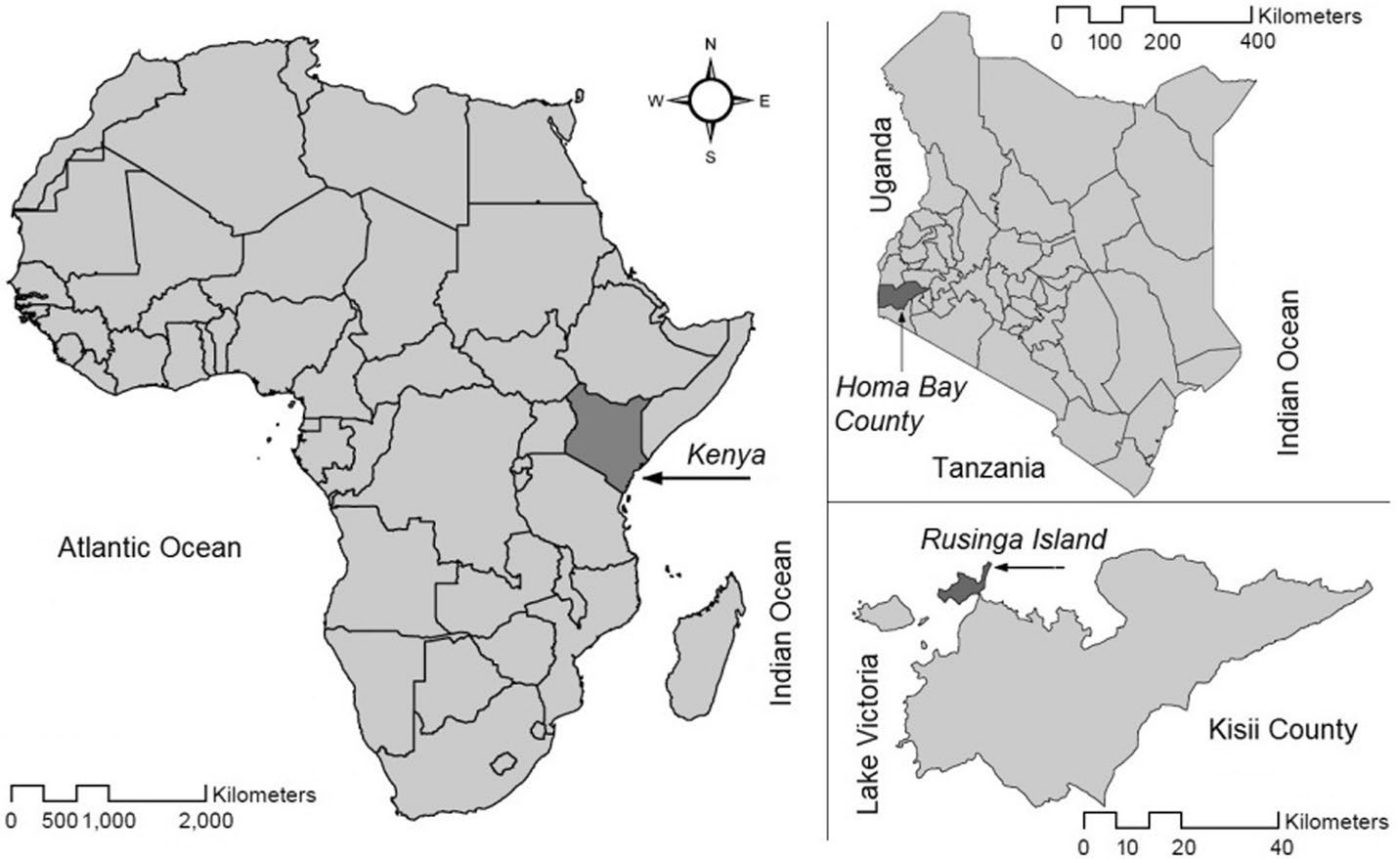
Study site: Africa with Kenya highlighted *dark grey*; in the *right upper corner* Kenya with Homa Bay County highlighted; Homa Bay County with Rusinga Island tinted in *dark grey*

Rusinga Island with approximately 25,000 inhabitants, is located in Lake Victoria, western Kenya (0°21′ S and 0°26 south, 34°13′ and 34°07′ east). The island is administratively part of Homa Bay county in western Kenya (Fig. 1) and is connected to the mainland with a causeway. The land surface area of Rusinga Island is approximately 44 km^2^ with an elevation between 1100 and 1300 m above sea level. Average daily temperatures lie between 16 and 34 °C with temperatures higher during the dry seasons which occur between June and October and late December–February. The SolarMal project, including HDSS activities, operates through the International Centre of Insect Physiology and Ecology (icipe) at the village of Mbita Point just across the causeway, on the mainland. The population of Rusinga Island belongs to the Luo ethnic community and, besides the national language of Swahili, DhoLuo is primarily spoken. Fishing and farming are the principal occupations. There are several health facilities in the area; one public health centre, three government-run dispensaries and three private clinics. A district hospital is found at Mbita Point. Malaria transmission occurs throughout the year, with peaks in transmission at the end of the rainy seasons where parasite prevalence is around 30 % (WHO Country Profile 2013: Kenya, Malaria). Furthermore, schistosomiasis, filariasis, HIV, and tuberculosis are endemic on Rusinga (Central Bureau of Statistics MoPaND. Kenya Demographic and Health Survey 2003).

### Data collection system

**Fig. 2:**
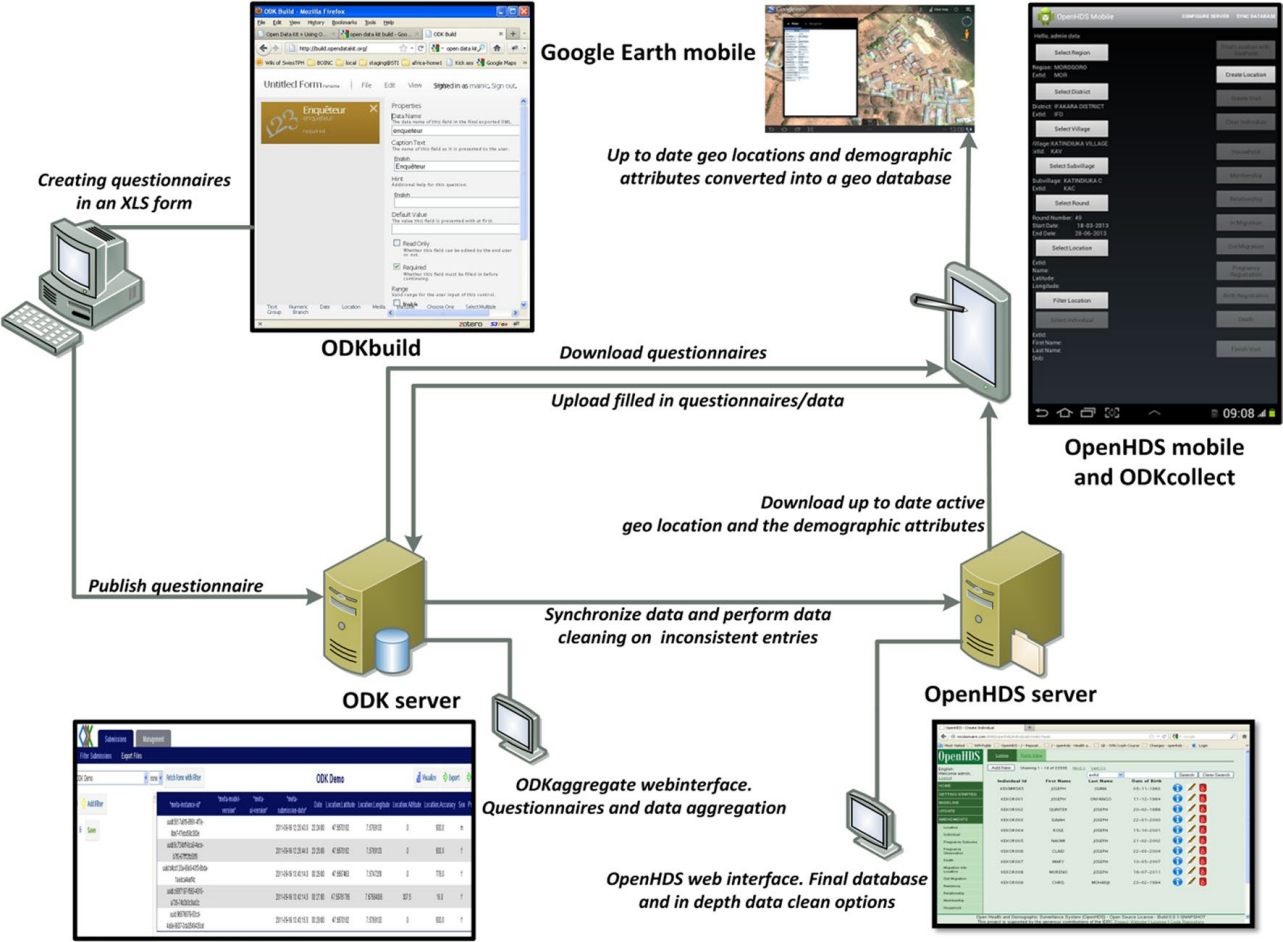
Data pathways using the ODK and OpenHDS platform: electronic questionnaires are created uploaded to the computer tablets by the ODK server. Wireless synchronization of digitalized data collected at the point of capture is transferred to the central data store based on the ODK server. Cleaned data is transferred to the OpenHDS server, that in turn synchronizes the up to date database to the computer tablets

The HDSS team consists of 10 fieldworkers (FWs), one fieldworker manager (FWM), a database manager and a system developer. Fieldworkers who spoke DhoLuo fluently and had a prior basic knowledge of computing were trained to use mobile tablet computer devices (Samsung Galaxy Tab 2, 10.1). A pilot study was conducted to test the usability of the computer tablets, as well as digital questionnaires, prior to the initial HDSS census. The HDSS uses the OpenHDS (Open Health and Demographic Surveillance) data system [15], a software platform that is based on a centralized database. This database is linked to a web application for data management, linked to a tablet computer-based mobile component which allows digitalization of data at the point of capture, and wireless synchronization to the central data store based on the Open Data Kit (ODK) platform [15, 19] (Fig. 2). ODK is a free, open-source application intended to facilitate mobile data collection services. ODK consists of two software components for data collection, transfer and storage, and various tools exist for the authoring of the electronic questionnaires used in the data collection process. ODK-Collect is used to render electronic questionnaire forms on mobile devices running the Android operation system, which includes forms to report core vital events as well as customized forms. ODK-Aggregate is a web application that supports data transfer and storage at a local server or a “cloud” server.

In addition to ODK-Collect, the OpenHDS mobile data collection application is installed on the tablets. This application contains a database which is pre-populated with data on the administrative location hierarchy in the study area (district, villages, neighbourhoods), and any information previously collected on individuals, houses and households in the area. This allows selection of the individual or house using the software during a visit to a household, and makes it possible to simply amend or add new information associated with the individual or house that has been selected. The differentiation made between houses and households follows the local culture, where the term *dhala* is used for a group that is socially and financially dependent or formed of related family members sharing the same facilities and recognizing one member as head of the household. A house is always defined as a single residential structure. The XLS-Form application is used for authoring questionnaire forms for ODK in the X-Form format. This allows integration of all possible structures of questions into the questionnaire: open answers, multiple choice answers, as well as posing constraints and requirements to answer outcomes. Questionnaires are published to ODK-Aggregate, and then downloaded to the tablets using ODK-Collect. This includes both questionnaires for capturing core vital events (births, deaths, in- and out-migrations) and study-specific questionnaires (parasitology, malaria incidence etc.). Electronic forms which are completed in the field using OpenHDS mobile are stored in ODK-Collect and synchronized over a Wi-Fi connection at the field station to the central database through ODK-Aggregate server (Fig. 2). After subsequent automated customized data checks, cleaned data is then submitted to the definite OpenHDS database. At the end of each update round, clean data is synchronized to the tablets to ensure that the most up to date information is taken back to the field for consecutive follow up surveys.

### Data collection rounds

The SolarMal project was initiated in January 2012 and will run through December 2015. The population census survey took place from June to September 2012, enumerating households, houses and individuals on the island. During the census survey, fieldworkers were assisted by individuals of the local community that are enrolled in a malaria programme, the Rusinga Malaria Project. The fieldworkers of the HDSS were familiarized with the population and geography of the island. In subsequent rounds of data collection, regular communication with the Rusinga Malaria Programme members and village elders enabled fieldworkers to find newly created households. All houses were mapped using the Global Positioning System function on the tablet, recording latitude and longitude with an accuracy of 5–15 m. Households are given a unique code consisting of two letters, relating to the name of the village where it is located, followed by a two digit number. Houses within a multi-house household have one extra letter, and all individuals are assigned a unique code comprising of five letters and two digits. Individuals were asked to provide their full name, sex, date of birth, main occupation and their relationship to the head of household. Subsequent analyses of individual data were performed using unique individual ID codes in order to ensure the anonymity of personal data.

**Fig. 3:**
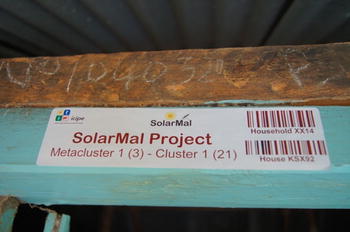
Project sticker with *barcode* on the doorpost of a house: Barcode scanning, integrated into the mobile data collection, allows quick identification of locations and study population to add or amend health and demographic information

To ensure that FWs are adding data to the correct corresponding house and individual in the field in subsequent follow up surveys, each house was provided with a door sticker showing its unique ID (Fig. 3). The unique ID is also expressed as a barcode which is scanned with the tablet on arrival at the house and recorded in the data base. Once scanned, the barcode is validated against existing barcodes in the mobile application of OpenHDS and the application allow questionnaires to be filled in and stored. Each household is visited three times a year to collect and update demographic and malaria-related data. Members of the HDSS team visit all residential structures in nine geographic areas on the island simultaneously taking approximately three months to cover their area. At all households observed pregnancies, new births, deaths and migrations which have occurred since the previous visit are recorded and updated. Digital questionnaires concerning demographic information are consistent with the HDSS questionnaire format of the INDEPTH network (See Table 1). Moreover, the standardized questionnaire formats are widely used in East Africa and Kenya and therefore apply well to our research site.

**Table 1:** An individual health questionnaire administered to everyone enrolled in the study

Question	Answer possibility
Individual ID	*ABCDE100*
Fieldworker ID	TO01
Illness over past 2 weeks	*Yes*; no
If illness reported: what symptoms?	(1) Diarrhoea, (*2*) *fever*, (3) vomiting, (4) rash, (5) bowel ache, (*6*) *head ache*, (7) cough/sore throat, (*8*) *joint pain*, (9) dizziness, (10) other (manually specify)
Fever over the last 2 days?	Yes; *no*
Current fever?	*Yes*, no
Under malaria treatment now?	Yes; *no*
If illness or fever reported: take temperature measurement	37.6
If temperature 37.4 °C or above: RDT test	(1) Negative, (*2*) *P. falciparum*, (3) other *Plasmodium*, (4) mixed malaria infection, (5) respondent refused to take test
Do you suffer respiratory symptoms?	*Yes*, no
If respiratory symptoms are experienced: Did you seek medical attention?	*Yes*, no
If medical attention: what medical attention was sought?	(1) Doctor, (2) nurse, (3) community health worker, (*4*) *traditional healer*, 5) other (manually specify)
Do you use any drug for the fever?	*Yes*, no
If using drugs against fever: which drugs?	(1) Anti malarials, (2) antibiotics, (*3*) *pain killers*, (4) other (manually specify)

In the right column an example of an individual’s answer in italics

Upon arrival at a household the barcode is scanned and a digital log, which includes the interview date and time, is automatically created. After recording deaths and births, migrations into or out of the household are documented. There is a differentiation between migrations within the island and from elsewhere. Individuals moving within the island maintain their individual ID which becomes associated with the new household. These individuals found in the system by filtering on their previous village and their name, subsequently selecting and migrating him or her. Moving out of Rusinga puts the individual in an inactive state in the database; people moving into Rusinga are provided with a new unique ID code if not previously enumerated, and all personal information is collected, as in the census survey. These individuals are found in the system by filtering on their previous village and their name and subsequently associating the individual ID with the new household ID through the completion of a migration form. If it is known that the individual in question does not plan to be a resident of the island no questionnaire is filled out. If it is known that an absent person is definitely coming back, no out migration is documented. To distinguish between temporary and permanent migration we use 6 months as a threshold. General information about the house construction, composition of household members and the presence and use of bed nets (as a malaria preventive tool) is collected for every house which is newly added to the database and for existing houses once per year.

### Use of geographical information

**Fig. 4:**
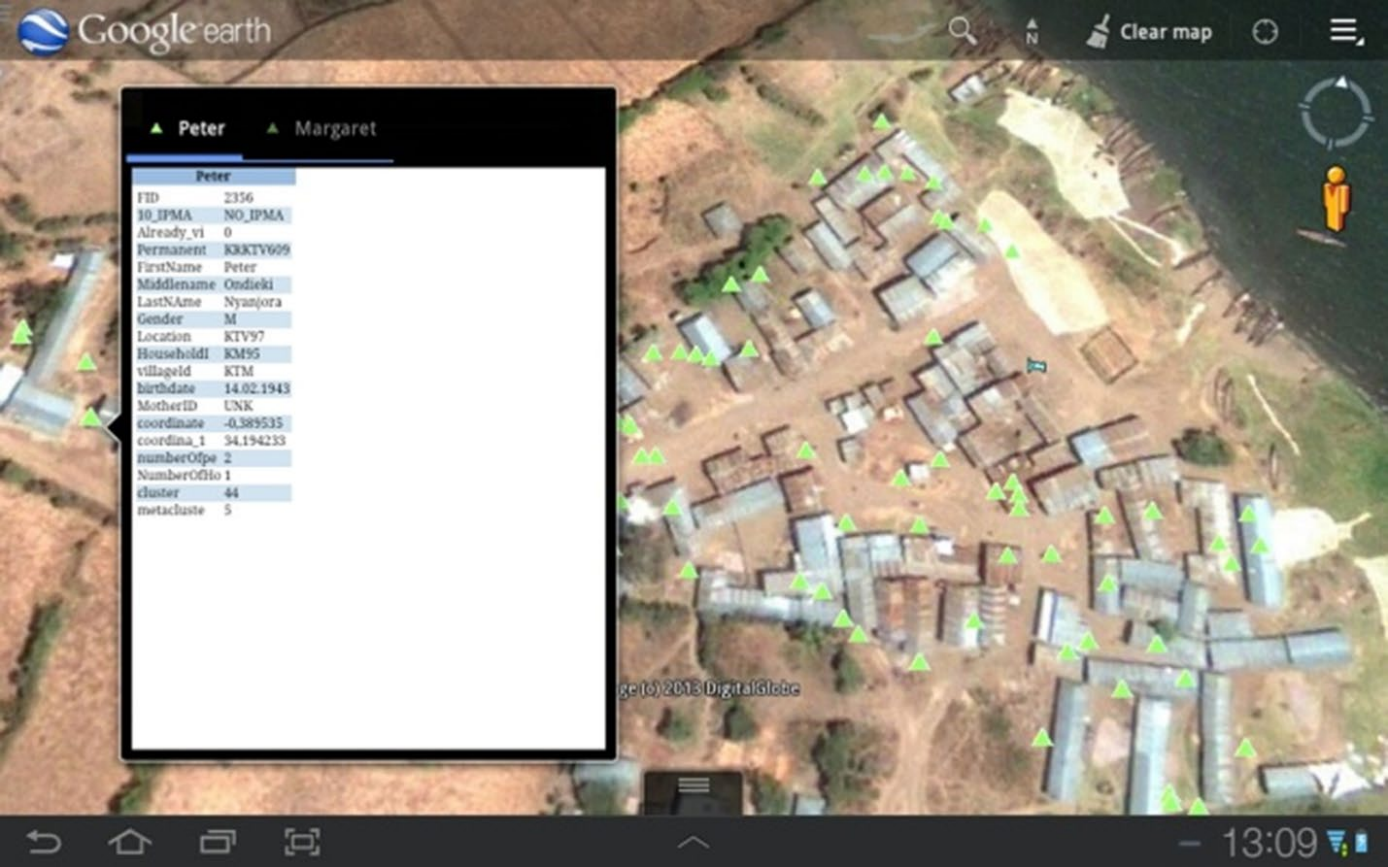
Navigating assigned houses: converting the up to date population database into a geodatabase displayed with Google Maps Mobile assists fieldworkers with tracking every house

On basis of the geographical coordinates of houses and demographic as well as malaria-related data gathered during the census of July 2012, the study design for the sequence of the rollout of the SolarMal intervention was developed and has been described elsewhere (Silkey et al., Personal Communications). Briefly, the island is divided into 81 clusters each containing 50 or 51 households, with nine clusters making up one metacluster. Metaclusters form the geographical basis for the HDSS follow up surveys. The fieldworkers are each assigned one of the metaclusters in which to visit every house and individual once during an interval of 3 months. One fieldworker is deployed to an area conditional on relative progress in the surveillance. For navigational purposes, the demographic database is converted into a geographic database (KML file), allowing us to plot houses to be visited in the Google Earth mobile (Version 7.1.3. 1255) application integrated in the tablet (constructed with ESRI 2011. ArcGIS Desktop: Release 09. Redlands, CA: Environmental Systems Research Institute). Using the GPS function, FWs can track themselves on the map navigating in real time from one house to another (Fig. 4). Furthermore, the geographic database also includes all server data enabling the FWs to select any house on the Google Earth map, consequently displaying the personal information of people living there.

### Data quality and management

Data quality is initially controlled by designing questionnaires which permit answers to fall within an acceptable range. For example, using input constraints a date can only be entered as a date format, only women can deliver a child, a body temperature must lie within 35–42 °C. After questionnaires have been entered in the field, the data is transferred to the ODK-Aggregate server. Unique IDs for individuals, houses and households are automatically generated per FW to ensure that no duplicate values are entered in the system. Questionnaires which were not fully completed are not accepted for upload to the server. Data is then transferred from ODK-Aggregate to the OpenHDS server using the Mirth Connect data integration platform [20]. All events entered during field visits are checked for inconsistencies during this step. Faulty records are filtered for further checking, and an error report is sent to the data manager by email. Births or deaths registered with an event date long in the past, multiple new-borns or separate deaths with the same date of event will be double checked with the FW or with the head of household. In addition, doubtful migrations are double checked, for instance if a child of 3 years old was found to be migrated because of marriage or work. Once in the OpenHDS server, the data manager has access to information about all individuals who have ever been active in the database, as well as their event history. A range of options to detect residual inconsistencies and perform data cleaning are available. An error often found in HDSSs is that individuals or households were duplicated during the census round under a slightly different name with different unique IDs at geographical border areas of FWs. An option to merge individuals and their past events provides a practical solution to this problem. In addition to this real time data quality control a web-based monitoring system was introduced that allows the data manager and FWM to extract a weekly snapshot of certain fieldwork related matters in the database [21]. The web interface displays information on where FWs have been in the past week, as well as which household visits are yet to take place. Subsequently, the geographical database converted to KML files are uploaded to tablets at the beginning of every follow up round. The tool automatically removes individuals and houses which have already been visited during a given round of surveillance from the visit plan, publishing a file with remaining houses to be visited that can be uploaded to the computer tablets. Furthermore, the tool can be used to produce graphs of how many individual and houses were visited and how many forms were filled in during the previous week, allowing the performance of fieldworkers to be tracked. The tool gives the opportunity to see where FWs have been, how long they have taken to conduct the work delivered, as well as which forms have been filled in and how often. This information gives the FWM a quick insight into every FW’s performance, so that inconsistencies can be addressed promptly and systematically. Additionally, on a weekly basis the tool generates 20 houses on basis of the houses already visited, to be revisited by the FWM. During re-visits, the usual procedure of demographic questionnaires is conducted and discrepancies between the results obtained by the FWM and FW are discussed with the FW in question.

Finally, all data of the HDSS, as well as entomological, parasitological, geographical and sociological data are fed into a MySQL relational database ready to be analysed. All data are linked through the unique individual, house or household IDs, making extraction of spatial and temporal data a mere case of entering the desired query into MySQL. Nightly backups of the databases are automatically copied to a network-attached storage system The local server is a highly secured drive located at the field station *icipe*.

### Ethical clearance

Ethical approval was obtained from the Kenyan Medical Research Institute (KEMRI); non-SSC Protocol No. 350. All participants are provided with information regarding the project outline, the ongoing HDSS procedures, the implementation of the intervention, and the collection and use of blood samples. Adults, mature minors and caregivers of children provided written informed consent in the local language agreeing to participation in the SolarMal project.

## Results and discussion

### Resource allocation

We describe a data collection and management platform which advances the electronic systems employed in HDSSs in developing countries a step further mainly by integrating mobile-device based data collection with a centralized real-time data system. This integration is one of the important improved aspects within the described HDSS, resulting in organizational and scientific advantages. HDSS sites often rely on paper-based conducting of questionnaires before the data is entered into a digital database [7, 9, 10, 22, 23]. The Android operating system is used on powerful tablet computers, allowing us to develop or deploy the desired software. In combination with the freely available mobile data collection software, ODK-Collect and OpenHDS mobile, collecting data on paper is set to become obsolete. This not only saves time because data can be entered by merely navigating through the digitalized form, and the process of double-entry of paper questionnaires into a digital format is no longer necessary. Fewer field workers and staff are required to perform the same job as before. Besides the cost-effectiveness on the basis of reduced staffing, the use of stationery is reduced to a minimum amount. Fieldworkers are provided with computer tablets, tablet protection covers and a paper notebook for occasional notes. Stationary in the office is reduced to a flip board to manage discussions, and some paper notebooks and pencils. All data collection and management is fully digital. Thus where traditional paper based HDSSs would approximately use one A4 for updates on household information and one A4 for individual health information, a digitalized data collection with 25,000 people and 8000 houses would save over 30,000 A4 papers per survey. In the last 5 years there are sites where HDSSs have migrated from paper-based to some sort of digitalized entering system [8, 24–27]. However, none of these sites have linked data collection software in the field directly to a real-time database. At the moment of writing, there is at least one other collection system using computer technology to integrate collection, management and database utilities; the LINKS system is in some ways similar to the system described in this paper [28]. LINKS also uses the ODK platform to collect data and is deployed at several sites in Africa. It is an easy implementable, cost reducing and efficient platform, however, the concept of a near real time database and its advantages seems not to be exploited. Furthermore, there are examples of health data collection systems where PDAs and telephones are used, which is considerably more efficient than the paper based surveillances. However, they show major limitations in terms of user-friendliness and scalability [29, 30]. This is mostly caused by the obsolescence and limited compatibility of software and hardware used.

### Time and organizational efficiency

Making use of the latest openly available technology, data collection in the field enables researchers and field workers to be time efficient, resulting in cost reductions and organizational efficacy. At most INDEPTH affiliated HDSS sites the Household Registration System (HRS) is used for managing demographic and health-related data, either by digitalizing filled in paper forms or direct digital entry in the field [8, 10, 22, 25, 26]. There are also examples of HDSS sites where a different data management system is developed relying on paper or non-paper based data collection [7, 9, 24]. The data collection system described in this paper has several advantages compared to the HRS in terms of organizational efficiency [31]: Firstly, traditional cleaning of data accumulating to an entity like an individual or household is largely removed. As the OpenHDS mobile application is a copy of the aggregated longitudinal database, in the application interface, adding data is only possible after selecting an existing entity. The constant uploading of collected data to the OpenHDS server and the synchronization of the database to the tablets makes reliable continuity of the data achievable.

Secondly, the entire process of creating an electronic questionnaire, up to viewing the collected data in a server, is a manageable, time efficient task for any scientist once basic training has been provided.

The XLS-Form authoring tool allows also non-computer scientists to create a questionnaire with the option to apply the preferred constraints. Concepts in questionnaires such as skip logic, input constraints, structured data model and an entry concept from the start, which the HRSs lack [31], have in our project let to only few forms of mistakes and errors that were relatively easy to detect. In a sample of our data we detected some incorrectly entered dates of birth and names, however in the following visit this personal data is always checked and corrected appropriately. The number of corrected mistakes in demographic data after one data collection round was never more than one percent. Simply uploading the XLS- form within ODK-Collect on the computer tablet allows one to conduct the questionnaires in OpenHDS mobile. All questionnaires related to the core demographic data collection are standardized and configured to OpenHDS mobile.

Thirdly, translating the real time database into a geographical database is a convenient way to assist FWs in real-time navigating their area of data collection. Demographic or disease-related data can be linked to a house location with its coordinate using the free Google Earth software. Tapping a house location on the device shows all the available household information. This combination of real time GPS navigation and fixed visiting points in space enables the FW to invest a minimal amount of effort in locating households at the study site. In this way fieldworkers of the HDSS manage to visit an average of approximately 15 houses and 40 people per day. The visiting of houses without a digital navigation platform can leave room for suboptimal walking routes.

Finally, after data collection has finished and data content has been cleaned, records can immediately be used to guide other parts of the project that rely on data collection structure of OpenHDS. Also, where the analysis of data in current HDSSs can only commence after it is manually entered and cleaned, this system allows one to have a dataset ready for analysis shortly after collection. Data cleaning is performed on a daily basis and, with roughly 500 data entries per day the data manager usually finishes routine cleaning in less than 2 h. Manually entering great amounts of questionnaires and post hoc cleaning of entered data can take many more hours even if every single questionnaire is digitally entered and cleaned in 1 min.

One aspect of this particular HDSS is the facilitation of healthy team cohesion. The SolarMal project is a multidisciplinary project with multiple researchers collecting data on sociological, entomological and parasitological outcomes integrated with a HDSS. The complete project data and storage is linked to the OpenHDS infrastructure, there are twice-monthly meetings with all project staff to discuss data-related issues and all research areas make use of the data gathered through the HDSS in planning and carrying out data collection activities and subsequently analysing the data.

### Data quality assurance

Organizational efficiency and data quality assurance go hand in hand, commencing from the OpenHDS platform where all data is centrally stored. Having the ODK-Aggregate and the OpenHDS server opens up the possibility for the data manager to check and clean the contents of data in a consistent way on a daily basis. This near-real-time quality assurance is conducted on the level of the ODK-Aggregate by means of a customized list of queries looking for inconsistencies that are easily detectable, like double visited individuals. The more in-depth data cleaning is then possible at the level of the OpenHDS. The platform offers a range of tools to check, research and amend all aspects of the demography in a population. Another large advantage of this system is the automatic generation of unique IDs. Automating the assignment of IDs avoids duplication of individuals or multiple individuals with the same ID. All data collected in the project are related to one of these three levels of unique IDs, in this way it is safeguarded that data collected is attributed to the right person or house. Furthermore, by means of the KML file, the FW knows which house is visited. Selecting the house ID in the OpenHDS mobile application directly gives access to editing and attaching new data to the individuals living there. Demographic and other questionnaires can easily be filled in and attached to the right unique ID, thus reducing confusing data accumulation drastically. In addition, all houses are provided with a door sticker with a unique bar code and the house and household ID. Scanning the barcode confirms the physical presence of the FW at the house, so that the data entered truly correspond to the house that is visited and it is not possible for a FW to enter data remotely. Lastly, a web-based monitoring of the database to monitor the performance of FWs is under development. This monitoring allows the FWs and data manager to follow the performance of every FW. Monitoring of fieldworkers to increase data quality is not a new concept [14, 15]. However, a near-real-time database that automatically displays FW performance is a convenience never described. Tracking the route walked by FWs, and observing the number of individuals and questionnaires filled in are currently the most prominent and helpful tools to detect fieldworker inconsistencies. More importantly, simple analysis of this data can shed light on interviewer bias, which can directly be discussed with the FW in question.

### Challenges and future research

Despite the advancement of and improved accessibility of information technology, the development and implementation of the described infrastructure in low and middle income countries will meet obstacles and limitations. Primarily, the requirement of electricity and a computer server near the field work site are vital. Likewise, this operation only becomes truly feasible with a trained data manager who has advanced I.T. skills. During this pioneering phase, having access to or collaborating with a software developer is also necessary. So, although on one hand cost and time savings are made in the long term, setting up the initial facilities requires a significant financial investment and demands a well-designed strategic plan for the context of the HDSS. Another complementary investment is the training of staff involved in the HDSS in how to handle the hardware and the software. Digitalization of the HDSS process from an existing paper-based system can lead to a drastic reduction of personnel, which facilitates the operational procedures of the HDSS.

Furthermore, there are many HDSS currently using paper based systems that desire to migrate to a fully digitalized HDSS. This transition can introduce a whole set of unforeseen difficulties that rely on complex logistical issues which necessitate more data and software professionals [32].

One of the biggest issues experienced throughout the past HDSSs, is dealing with migration of the population under study. Where the OpenHDS system allows this problem to be handled much more promptly than paper-based or obsolete household registration systems, it is still a challenge to make sure that internal migrations between households are correctly processed. Individuals can always be immigrated again, but the reintroduction relies on the name given by the person in question. We experienced that sometimes other names are given or the original name was incorrectly provided.

## Conclusion

In regions lacking adequate organization to monitor demographic and health information little is known about population dynamics and the epidemiology of disease. It is these areas where health is often heavily compromised and where collection of specific health-related data can greatly improve our understanding of health issues. The HDSS within the SolarMal project provides an example of a user-friendly infrastructure for field data collection in evidence-based research in low and middle income countries by making use of the currently available technologies. Whereas most HDSSs still work with paper based or obsolete digital systems, this paper describes a totally digitalized platform that allows fieldworkers and field managers to quickly and systematically keep clean data, make fewer mistakes with data collection and make use of a structured data model and entry concept from the start. Stakeholders such as government health officers, local administrators and scientists have easy access to real time data storage on a secure central database which enables them to conduct near-real-time quality assurance. Besides, remote progress monitoring allow scientists to quickly detect inconsistencies. Most importantly, this system could radically increase cost-effectiveness by saving time and money on stationery, data clerks, organizational costs and manual logistics.
